# A System of Surgery

**Published:** 1896-01

**Authors:** 


A System of Surgery. In a Series of Contributions by twenty-five English Authors. Edited by Frederick Treves, F. R. C. S., Surgeon to, and Lecturer on, Surgery at the London Hospital; Examiner in Surgery at the University of Cambridge. In two large octavo volumes. Volume I., 1178 pages, 463 engravings and two colored plates. Cloth, $8.00. Philadelphia: Lea Brothers & Co., Publishers. 1895.

The aim of this work is to present in an authoritative and concise way the pathology, clinical manifestations and treatment of the various surgical conditions with which the surgeon has to deal, and this is accomplished in a way which we cannot but think will prove most satisfactory both to the surgeon and student. No attempt is made to go into minute details of different operations, but the underlying principles of treatment in general are given fully and plainly. Ophthalmic surgery is entirely excluded from the work, it being regarded as too important and comprehensive to be confined within necessarily limited compass.

The various subjects are treated by acknowledged authorities in the same and include such familiar names as those of Watson Cheyne, Barker, Lane, Morris, Gould, Sutton, Bowlby and many others, thus insuring for each subject that it is written in a thorough and practical manner and contains the results of mature experience and judgment. The subjects treated include surgical bacteriology and pathology, minor surgery, injuries and diseases of the various tissues of the body, constitutional conditions with surgical sequelæ and other chapters upon important subjects.

One chapter upon the influence of constitutional conditions upon injuries, written by the editor, is excellent and should invite others to further investigation along these lines. This chapter is quite characteristic of the book as a whole in that it is so eminently practical. The illustrations are mostly original and many of them elucidate the text in a very satisfactory way. The publishers have given to the profession a book quite up to the high standard of excellence for which they have long been noted.

